# A multiorgan map of metabolic, signaling, and inflammatory pathways that coordinately control fasting glycemia in mice

**DOI:** 10.1016/j.isci.2024.111134

**Published:** 2024-10-11

**Authors:** Florence Mehl, Ana Rodríguez Sánchez-Archidona, Ida Meitil, Mathias Gerl, Céline Cruciani-Guglielmacci, Leonore Wigger, Hervé Le Stunff, Kelly Meneyrol, Justine Lallement, Jessica Denom, Christian Klose, Kai Simons, Marco Pagni, Christophe Magnan, Mark Ibberson, Bernard Thorens

**Affiliations:** 1 Vital-IT Group, SIB Swiss Institute for Bioinformatics, 1015 Lausanne, Switzerland; 2 Center for Integrative Genomics, University of Lausanne, 1015 Lausanne, Switzerland; 3 Université de Paris Cité, BFA, UMR 8251, CNRS, 75013 Paris, France; 4 Lipotype GmbH, Dresden, Germany

**Keywords:** Bioinformatics, Omics, Physiology, Transcriptomics

## Abstract

To identify the pathways that are coordinately regulated in pancreatic β cells, muscle, liver, and fat to control fasting glycemia we fed C57Bl/6, DBA/2, and Balb/c mice a regular chow or a high fat diet for 5, 13, and 33 days. Physiological, transcriptomic and lipidomic data were used in a data fusion approach to identify organ-specific pathways linked to fasting glycemia across all conditions investigated. In pancreatic islets, constant insulinemia despite higher glycemic levels was associated with reduced expression of hormone and neurotransmitter receptors, OXPHOS, cadherins, integrins, and gap junction mRNAs. Higher glycemia and insulin resistance were associated, in muscle, with decreased insulin signaling, glycolytic, Krebs’ cycle, OXPHOS, and endo/exocytosis mRNAs; in hepatocytes, with reduced insulin signaling, branched chain amino acid catabolism and OXPHOS mRNAs; in adipose tissue, with increased innate immunity and lipid catabolism mRNAs. These data provide a resource for further studies of interorgan communication in glucose homeostasis.

## Highlights

• Mice from three mouse strains were fed with an RC of HFD for three periods of time
• Physiological and multi-tissue transcriptomic and lipidomic analysis were performed
• Data fusion analysis identified tissue-specific pathways associated with fasting glycemia
• A map of the recruited metabolic, signaling, and inflammatory pathways is provided

## Graphical abstract

**Figure:**
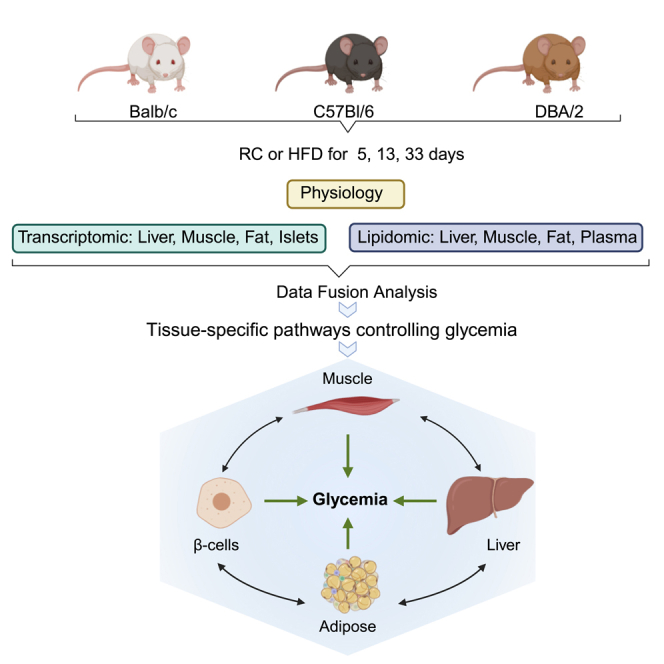

## Introduction

The balance between insulin secretion by pancreatic islet β cells and insulin action on muscle, liver, and fat is essential to control glycemic levels. Studies over the past decades have led to the description of the main pathways that control glucose-stimulated insulin secretion (GSIS) by β cells and insulin signaling in muscle, liver, and fat.

GSIS is triggered by the Glut2-glucokinase (Gck)-K_ATP_ channel pathway, which induces membrane depolarization leading to Ca^2+^ entry and Ca^2+^-dependent insulin granule exocytosis. This pathway is amplified by glucose metabolism-derived coupling factors and is modulated by a plethora of signals reflecting the metabolic state of the organism. These signals are nutrients such as amino acids or free fatty acids^1^; various hormones including the gut-derived glucoincretins GLP-1 and GIP^2^; neurotransmitters of the autonomous nervous system^3^; and chemokines and cytokines produced by immune cells and adipocytes.^4^^,^^5^^,^^6^ Secretion is also strongly influenced by interactions of β cells with the extracellular matrix (ECM) and with neighboring cells through cell adhesion molecules^7^^,^^8^^,^^9^^,^^10^^,^^11^^,^^12^ and gap junctions.^13^^,^^14^

Insulin action is initiated by its binding to the insulin receptor (Insr) followed by the recruitment of insulin receptor substrates (Irs) and activation of the Pi3k/Akt or of the Ras/Raf/Map kinase pathways.^15^ In adipocytes and muscle, the Pi3k/Akt pathway increases Glut4 cell surface expression and, consequently, glucose uptake and metabolism.^16^ In muscle glucose can be stored as glycogen or triglycerides (TGs) or used through the glycolytic pathway, the Kreb’s cycle, and the OXPHOS chain to form ATP to fuel muscle contraction. In white adipocytes, glucose is mostly converted to glycerol 3-phosphate to esterify free fatty acids for storage as TGs. In liver, glucose is taken up by Glut2 and is phosphorylated into glucose-6-phosphate (G6P) by glucokinase, whose expression is controlled by insulin. G6P is then directed toward glycogen synthesis, or to the pentose phosphate shunt and glycolytic pathway to generate NADPH, acetyl-CoA and ATP to fuel lipogenesis.

Insulin signaling efficacy can be reduced by multiple mechanisms which, when over activated cause the insulin resistance that characterizes obesity and type 2 diabetes (T2D).^17^ These mechanisms include a downregulation of INSR cell surface expression and the phosphorylation of the INSR and IRSs by Ser/Thr kinases activated by metabolic or inflammatory signals.^15^ Insulin resistance is also associated with reduced OXPHOS activity,^18^ decreased branched chain amino acid (BCAA) degradation^19^ and increased oxidative stress.^20^^,^^21^ Tissue inflammation, characterized by the presence of cells of the innate immunity and the production of chemokines, cytokines or interleukins, is also causally linked to the development of insulin resistance.^20^^,^^22^

Dysregulations of the pathways that control insulin secretion or insulin action frequently leads to development of hyperglycemia. This has been demonstrated in innumerable gene knockout mouse studies. For instance, knocking out *Gck* in pancreatic β cells,^23^ *Glut4* in muscle,^24^ glucose-responsive transcription factor *Chrebp* in adipose tissue,^25^ or the *Insr* in hepatocytes^26^ all lead to hyperglycemia. Additionally, the diabetic phenotype of wild type or gene knockout mice is often exacerbated by the metabolic challenge of a high fat diet (HFD). Although such studies have been used to highlight the role of selected genes and pathways on whole body glycemic control, they did not describe how the activity of these pathways is coordinately regulated among various tissues to control glycemia.

Here, we wished to identify the pathways that are regulated in a tissue-specific manner to control fasting glycemia, a parameter that is diagnostic of normal glucose homeostasis or of the appearance of pre-diabetes or overt diabetes.^27^^,^^28^ To this end we investigated selected key organs that control glucose usage and production (pancreatic islets, soleus muscle, liver, and visceral white adipose). We used mice with different genetic backgrounds and sensitivity to a HFD-induced metabolic stress and analyzed them when their glycemic levels were still in the physiological range. We reasoned that the integrated transcriptomic analysis of these tissues across all experimental conditions could allow the identification of the signaling or metabolic pathways that are most tightly regulated with, and may control glycemic levels. Such approach requires that a relatively large number of mice, displaying a range of glycemic levels, are studied so that association between tissue-specific gene pathways and phenotype (glycemia) can be meaningfully identified.

We fed C57Bl/6, DBA/2, and Balb/c mice for 5, 13, and 33 days with a regular chow (RC) or a HFD, which triggers a strong transcriptomic adaptation in many organs but had limited effects on glycemia over the experimental periods used. We performed RNA-seq analysis of islets, soleus muscle, liver and visceral fat and lipidomic analysis of soleus muscle, liver, visceral fat, and plasma. We then used a data fusion approach to identify organ-specific co-expression modules that could explain the variation in glycemic levels across all mouse strains and feeding conditions. This led to the identification of pathways that may be coordinately recruited in each of the investigated organs for the physiological control of fasting glycemia.

## Results and discussion

### Mice and omics analysis

**Figure 1:**
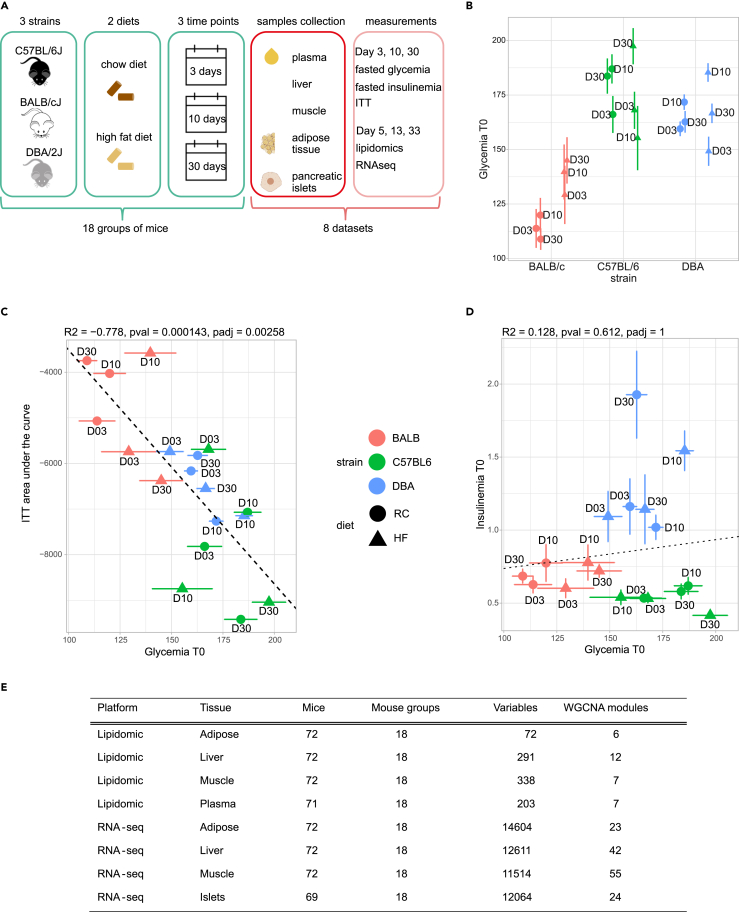
Experimental design, mouse physiology and multi-omics analysis (A) Scheme of the experimental design. (B) Five-hour fasted glycemia in Balb/c, C57Bl/6 and DBA/2 mice fed an RC or HFD. Each data point represents the mean ± SEM (*n* = 10–12 mice) glycemic level of the mice at 3, 10, and 30 days of diet feeding. Circles: RC fed mice; triangles: HFD fed mice. (C) Correlation between 5-h fasted glycemia and the area under baseline of an insulin tolerance test. Each data point represents the mean ± SEM (*n* = 10–12 mice) for each time point, strain and feeding condition. (D) Correlation between basal insulinemia and basal glycemia indicating no significant correlation. Each data point represents the mean ± SEM (*n* = 10–12 mice) for each time point, strain and feeding condition. (E) Summary table of the number of mice, mouse groups, variables, and WGCNA modules for each each omics platform used and tissue analyzed.

To investigate the interorgan interactions that control basal glycemia, we used C57Bl/6J, DBA/2, and Balb/c mice, which are characterized by markedly different metabolic adaptations to an HFD feeding with distinct effects on insulin secretion and action.^29^^,^^30^ Eight-weeks-old mice were fed with an RC or an HFD and body weight, basal glycemia and insulinemia were measured at 2, 10, and 30 days (Figure 1A, Table S1). Five-hour fasted (basal) glycemia were lower in RC fed Balb/c mice than in the C57Bl/6 and DBA mice and, upon HFD feeding, basal glycemia increased in Balb/c mice but not in the two other strains (Figure 1B). These differences in glycemic levels were exploited to identify molecular pathways associated with the regulation of fasting glycemia in all conditions studied, regardless of inter-strain variations (see further text). Basal glycemic levels were negatively correlated with insulin sensitivity assessed in i.p. insulin tolerance tests (ITT) (Figure 1C) but were not correlated with 5-h fasted insulinemia (Figure 1D). These data suggest that insulin sensitivity can explain the basal glycemic levels. In contrast, basal insulin plasma levels remained constant with increasing glycemia, suggesting a relative defect in insulin secretion, although differences in insulin clearance could also contribute to this observation.

### A global statistical model integrating -omics data predicts basal glycemia

The mice were euthanatized at 5, 13, and 33 days for RNA-seq analysis of liver, visceral white adipose tissue, soleus muscle, and pancreatic islets and for lipidomic analysis of liver, visceral adipose tissue, soleus muscle, and plasma (Figures 1A and 1E). One RNA-seq dataset for each organ was generated from 72 mice (liver, adipose, muscle) or 69 mice (islets) belonging to 18 mouse groups (3 mouse strains x 2 diets × 3 three time points; for each group data are the mean of 2–4 mice); one lipidomics dataset was generated for the liver, the muscle, adipose, and plasma from 71 to 72 mice (partially overlapping with the 72 previous mice) (Figure 1E).

The -omics data were integrated into a global model to predict basal glycemia using a data fusion approach (see STAR Methods for details). This approach enabled us to first assess whether the combined -omics data could be used to predict basal glycemia, and second, if so, to measure the contributions of each dataset to the prediction. The starting point for the analysis was a set of data tables (or blocks) representing either gene expression or lipid concentrations across samples in the different tissues. Since each of the data blocks have different dimensions that could lead to biases in the modeling, it was necessary to first reduce the number of dimensions for each block. For this we used WGCNA^31^ on both the transcriptomics and lipidomics data, reducing their dimensions to a smaller number of gene or lipid modules (Figure 1E). Each of these modules represents sets of mRNAs or lipids that show similar coexpression patterns within a tissue and can be summarized using an eigengene.^31^ Initially, we integrated the mRNAs and lipid eigengenes for each tissue into an unsupervised model using common dimensions.^32^ This method is similar to principal-component analysis (PCA), except that the samples are projected into common dimensions derived from integrated mRNAs and lipid data. This model enabled us to assess the main sources of variation in the data independently of any particular outcome variable. The results of this analysis (Figure S1) show good separation of strains and to a lesser extent diets in the first two dimensions, indicating that mouse strains and diets explain most of the overall variation in the integrated dataset.

We then built a multiblock prediction model by integrating the gene and lipid eigengenes with basal glycemia as the outcome using a regression-based multivariate modeling method, Consensus OPLS (consensus orthogonal projection to latent structures^33^). This supervised data fusion approach attempts to segregate samples according to an outcome (in this case basal glycemia) along the first dimension (x axis in Figure S2A), with variation not related to the outcome in the y axis. Using this model, we identified the gene and lipid tissue modules that best explain basal glycemia across all strains and feeding conditions. The results show separation of mouse samples according to basal glycemia along the x axis of the score plot (Figure S2A). The model showed good prediction compared to random data based on a permutation test (Figure S2B). The gene and lipid modules were then ranked according to a score (VIP; variable importance in projection) that captures how much each module contributes to basal glycemia. These ranked modules were then further investigated to identify biological pathways in each tissue that could be involved in the control of basal glycemia.

### Pathway analysis of transcriptomic data

**Figure 2:**
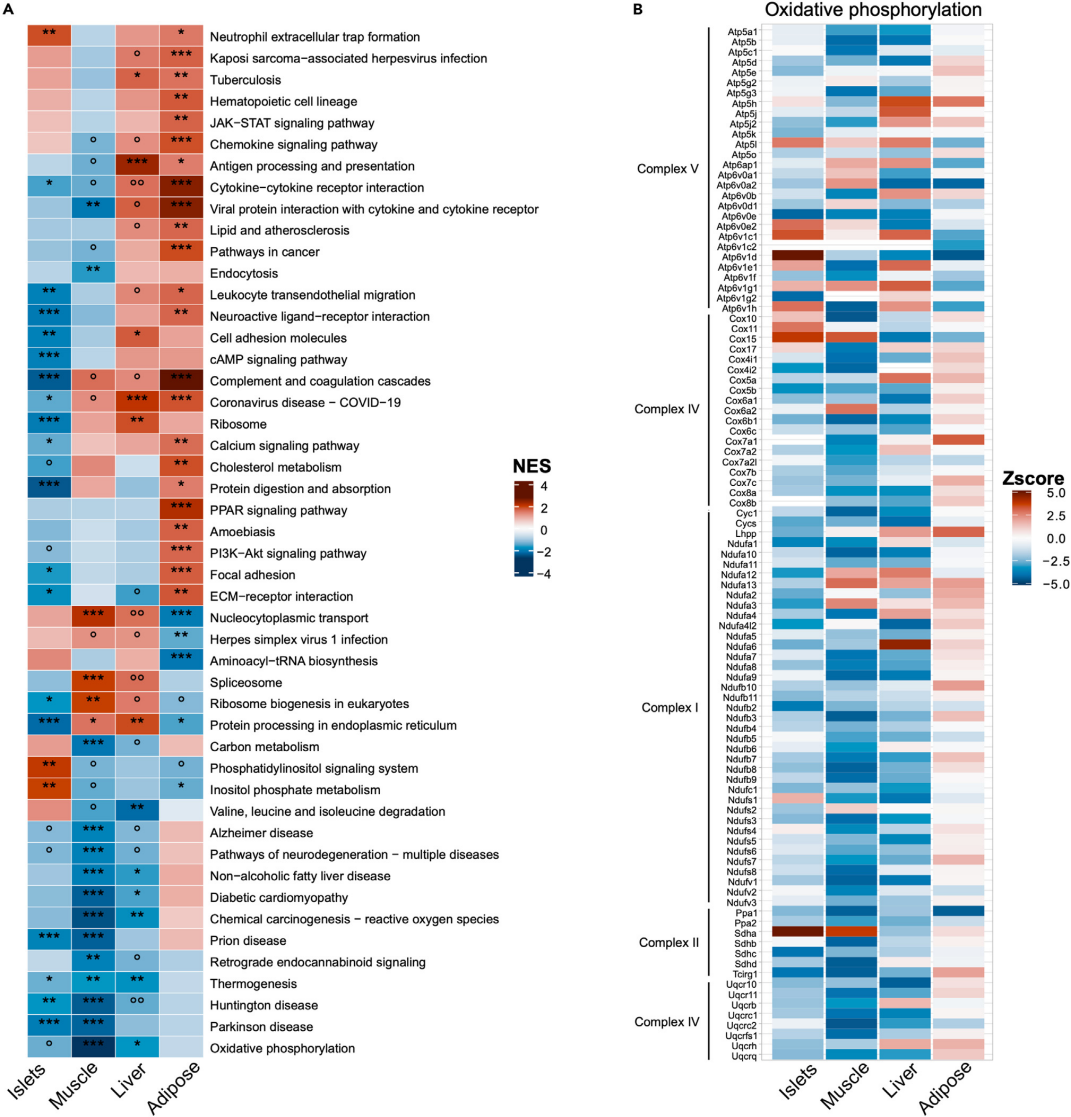
Heatmap of KEGG terms regulated in each tissue (A) Heatmap of normalized enrichment scores (NES) of 48 KEGG pathways across the four tissues. Pathways were selected if significantly enriched (adjusted *p* value ≤0.01) in at least one tissue. ∗ adjusted *p* value <0.05, ∗∗ adjusted *p* value <0.01, ∗∗∗ adjusted *p* value <0.001, ° *p* value <0.05, °° *p* value <0.01, °°° *p* value <0.001, no symbol *p* value >0.05. (B) Heatmap of the expression (Z-scores) of the OXPHOS mRNAs comprising the Oxidative phosphorylation KEGG term across the four investigated tissues.

To search for pathways that are modulated with basal glycemia, a gene set enrichment analysis (GSEA) on Kyoto Encyclopedia of Genes and Genomes (KEGG) database was run for each tissue with a gene list ranked on a *Z* score value (see STAR Methods for details). This score captures the relationship between a gene and the co-expression module as well as its relationship to the phenotypic trait. This *Z* score is computed to maximize the signal/noise ratio. To compare how pathways are regulated across tissues, the list of all enriched terms across all tissues was restricted to the terms enriched with an adjusted *p* value ≤0.01 in at least one tissue, leading in a list of 48 terms (Figure 2A). This heatmap shows that the pathways that were significantly up or downregulated were often common to two or more tissues, and the direction of regulation were either the same or opposite across tissues. One example is the “Oxidative phosphorylation” module, which is downregulated in islets, liver, and muscle; it is, however, not significantly regulated in adipose tissue. This module is enriched in OXPHOS mRNAs, which also comprise most of mRNAs of the “Thermogenesis”, “Huntington disease”, “Parkinson disease”, and “Prion disease” modules. The tissue-specific expression of the OXPHOS mRNAs is illustrated in the heatmap of Figure 2B. Further, we will discuss how the tissue-specific regulation of the identified pathways and of their mRNAs are related to the control of fasting glycemia.

### Tissue-specific pathways related to fasting glycemia

#### Pancreatic islets

**Figure 3:**
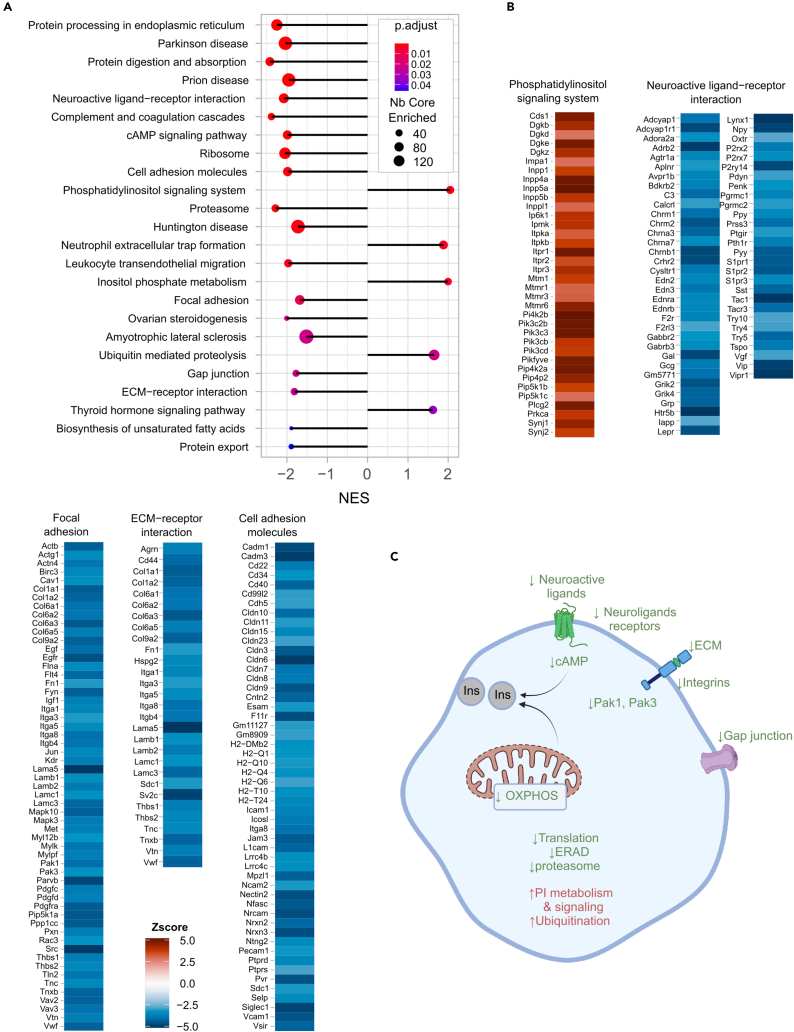
Pathways regulated with glycemia in pancreatic islets (A) Plot showing the major KEGG pathways regulated with glycemia in pancreatic islets. The negative and positive NES values indicate, respectively, down- or up-expression of the indicated terms with glycemia. The colors indicate the *p* values of the enrichment score, and the size of the dots, the number of genes in each term. (B) Illustration of the mRNAs that comprise selected KEGG terms with the color indication of their *Z* score value. (C) Scheme of the major pathways down- (green) and up- (red) regulated with glycemia and that are involved in the control of β cell function.

The pathways that were up-or down-regulated with fasting glycemia in islets are shown in Figure 3A. Three terms related to cellular proteostasis were downregulated: the “Ribosome”, the “Protein processing in the endoplasmic reticulum” and the “Proteasome” terms (Table S2). These included, respectively, 14 cytoplasmic and mitochondrial ribosomal genes, suggesting reduced translational activity; 47 mRNAs encoding proteins involved in endoplasmic reticulum (ER) protein processing, 16 of which are part of the ER-associated protein degradation (ERAD) pathway; 17 mRNAs encoding proteasome subunits. In contrast, the “Ubiquitin mediated proteolysis” term, which includes 31 mRNAs encoding components of the E1, E2, and E3 ubiquitin transfer system was upregulated (Figure 3B).

Cellular proteostasis in β cells plays a critical role in preserving insulin biosynthesis and secretory activity^34^ and decrease in proteasome activity reduces GSIS by reducing the activity of the K_ATP_ channel and of the voltage-dependent Ca^++^ channel.^35^^,^^36^ Increased expression of ubiquitination mRNAs when the proteasomal ones are downregulated suggests increased non-degradative ubiquitination. This process contributes to optimal beta-cell function through the control of mitophagy activity^37^ or the expression and activity of several transcription factors that regulate beta-cell differentiation and function.^38^^,^^39^

Other downregulated pathways were the “Parkinson disease”, “Huntington”, and “Amyotrophic lateral sclerosis” terms, which are highly enriched in OXPHOS genes (Figure 2B). Decreased OXPHOS activity and ATP production also reduce GSIS. Thus, the basal insulin plasma levels that remained stable with increased basal glycemia (Figure 1D) were associated with reduced islet protein biosynthesis, ERAD, and proteasome and OXPHOS activities. Interestingly, when the same data fusion analysis was performed separately with data from RC or HFD fed mice, these same pathways were identified using only the RC fed mice dataset (Figure S3A); this suggests that the activity of these pathways is predominantly determined by the mouse genetic background.

The other downregulated pathways (Figure 3A) not only appeared in the combined analysis of RC and HFD mice but also when the analysis was performed only with the data from the HFD mice (Figure S3B), suggesting that they were regulated by the metabolic stress of the diet. Strikingly, the downregulated “Neuroactive ligand receptor interaction” term included 32 hormone receptors, and receptors for GABA, ATP, acetylcholine and adrenaline (Figure 3B). This indicates reduced sensitivity of the beta-cells to cues generated by multiple organs, which inform the β cells on the organism’s metabolic state.^40^^,^^41^ The downregulation of the “cAMP signaling” term, which included mRNAs for adenylate cyclases (*Adcys*), G protein subunits (*Gnai3*, *Gnas*), phosphodiesterases (*Pdes*), and the transcription factor *Creb3l4* (Figure 3B) further support a decreased activity of the aforementioned receptor intracellular signaling. Interestingly, the “Biosynthesis of unsaturated fatty acids” term consists of mRNAs for several desaturases and elongases (*Scd1*, *Scd2*, *Fads1*, *Fads2*, *Elovl1*, *Elovl2*, *Elovl4*, *Elovl6*), which generate various lipids that control β cell mass and insulin secretion.^29^^,^^42^^,^^43^

There was also an important downregulation of the “Focal adhesion”, “ECM receptor interaction”, and “Cell adhesion molecules” terms (Figure 3B), which included mRNAs encoding extracellular matrix proteins (including collagen subunits, laminins and neurexins), cell adhesion molecules (cadherins), integrins, and the integrin signal transducers *Pak1* and *Pak3* (Figure 3B). Expression of the mRNAs encoding the gap junction proteins *Gja1* (connexin 43) and *Gjd2* (connexin 36) was also downregulated (see “Gap junction” term Table S2). Interaction of the β cells with their extracellular matrix and with other islet cells through cadherins and gap junctions preserve β cells’ architecture and electrical coupling required for optimal insulin secretion capacity.^12^^,^^14^^,^^44^^,^^45^^,^^46^

Two interesting terms were upregulated, the “Phosphatidylinositol signaling system” and “Inositol phosphate metabolism” terms, which comprise mostly the same mRNAs. Figure 3B shows the heatmap of the “Phosphatidylinositol signaling system” mRNAs. They included increased expression of 4 diacylglycerol kinases (*Dgks*), which reduce the intracellular levels of diacylglycerol and, thus, PKC activity; higher expression of inositol-phosphate phosphatases (five *Inpp* and four myotubularin: *Mtm1*, *Mtmrs*) suggesting increased degradation of inositol phosphates. On the other hand, there was increased expression of two inositoltetrakisphosphate kinases (*Itpks*) and three subunits of the inositol-phosphate receptor (*Itprs*), as well as increased expression of the phosphatidylinositol-kinases *Pikfyve*, and of several *Pik3s*, *Pip4ks*, and *Pip5ks.* Together these observations suggest increased phosphatidylinositol turnover with increased glycemic levels. The effect on insulin secretion is uncertain and would require measuring the concentrations of inositol phosphate species in islets to determine their contribution to PKC activity or Ca^2+^ release from the ER, two important mechanisms regulating GSIS.^47^

Collectively, these observations suggest that OXPHOS activity and proteostasis fine-tune β cells secretory activity in RC fed mice (Figure 3C). When mice are fed an HFD a multitude of hormonal and neuronal signals were downregulated with increased glycemia, as were mRNAs involved in free fatty acid elongation and desaturation and in the interaction of the β cells with the ECM and with neighboring cells. These observations, thus, support the hypothesis that the constant insulinemic levels measured irrespective of blood glucose concentrations indicate a relative decrease in insulin secretion capacity. It is striking, however, that major genes involved in GSIS, such as *Glut2*, *Gck*, the K_ATP_ channel subunits *Kir6*_*2*_ and *Sur1*, and the voltage-gated Ca^++^ channel, which are regulated in diabetic conditions^48^ and are diabetes susceptibility genes,^49^ were not differentially expressed with changes in basal glycemia. There was also no association of glycemia with a differential expression of β cell differentiation markers (*Pdx1*, *NeuroD*, *Pax4*, *Slc16a1*, *Ldh*, *Aldh1*^50^^,^^51^ or lipid modifying enzymes that participate in the control of GSIS (*Abhd6*, *Cpt1*).^1^ This highlights the importance of the interactions of β cells not only with the global internal milieu, but also with the ECM and with adjacent β cells through gap junctions in the control of basal insulinemia.

#### Soleus muscle

**Figure 4:**
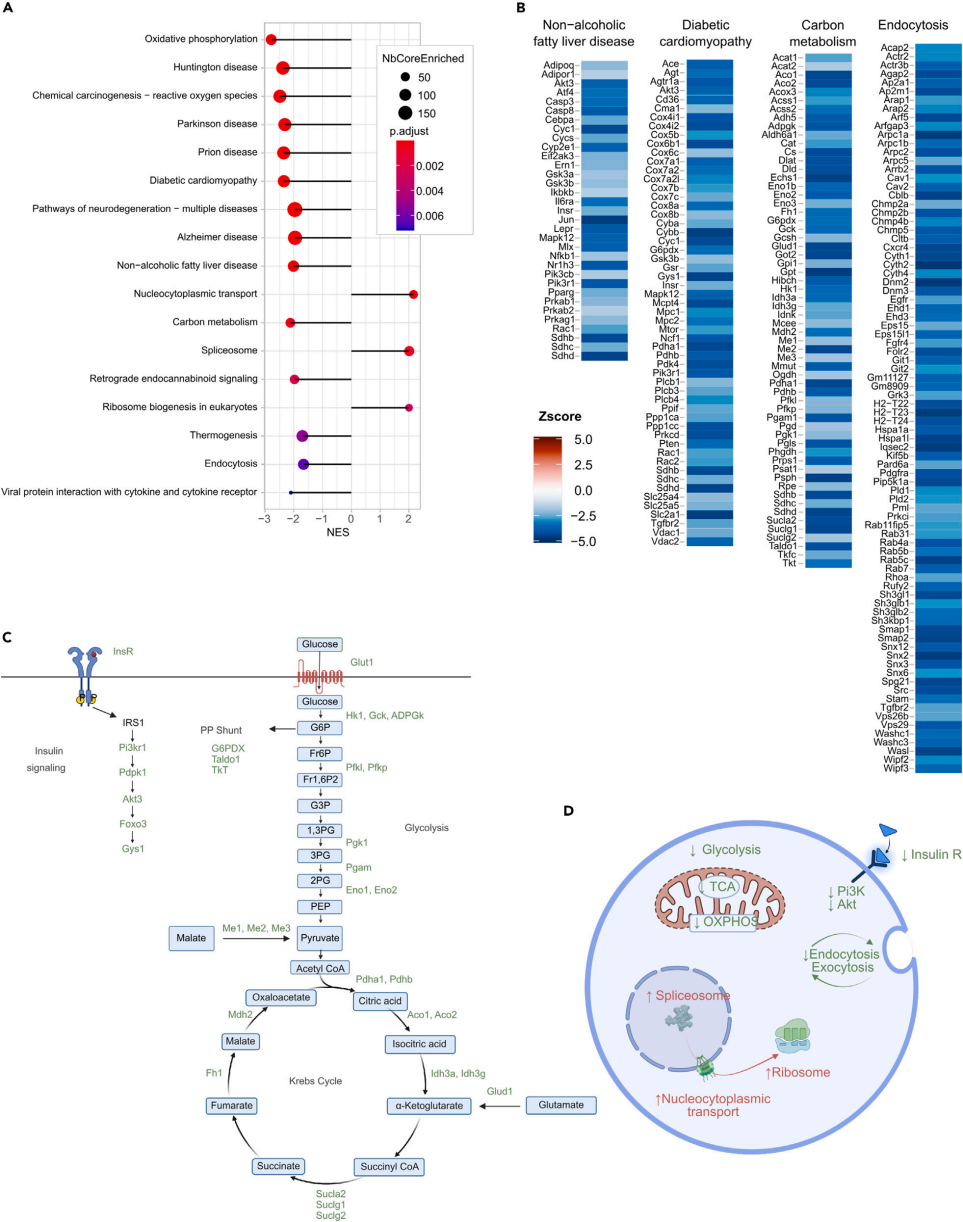
Pathways regulated with glycemia in soleus muscle (A) Plot showing the major KEGG pathways regulated with glycemia in muscle. (B) Illustration of the mRNAs that comprise selected KEGG terms with the color indication of their *Z* score value. (C) Scheme of the insulin signaling pathway, and of the glycolytic, pentose phosphate, and Krebs’ cycle pathways with, in green, the mRNAs whose expression is downregulated with glycemia. (D) Scheme of the major down and upregulated pathways that are involved in the control of muscle function.

In soleus muscle three terms were up regulated with glycemia (Figure 4A). The “Spliceosome” term contained 43 mRNAs, 39 of which are involved in pre-mRNA splicing; the “Nucleocytoplasmic transport of mRNA” term contained 43 mRNAs, 14 of which code for nuclear pore complex proteins and most of the other mRNAs encode proteins involved in nuclear protein import or export; the “Ribosomes biogenesis” term comprised 29 mRNAs encoding proteins controlling ribosomal RNA production, and the assembly of the large and small ribosomal subunits (Table S3). These observations suggest higher rates of premRNA maturation, mRNA export to the cytoplasm, and ribosome production, which together suggest that protein biosynthesis activity increases with higher blood glucose concentrations.

The downregulated terms included “Oxidative phosphorylation”, “Thermogenesis”, “Huntington disease”, “Parkinson disease”, “Prion disease”, and “Alzheimer’s disease”, which mainly comprised mRNAs encoding subunits of Complex I, II, III and IV of the electron transport chain and of the ATP synthase (Figure 2B). The “Diabetic cardiomyopathy”, “Non-alcoholic fatty liver disease” and “Chemical carcinogenesis—reactive oxygen species” terms included, in addition to OXPHOS mRNAs, several mRNAs encoding key components of the insulin signaling pathway: *InsR*, *Pik3r1*, *Akt3*, *Pdpk1*, *Gys1*, *Foxo3*, and *Glut1* (Figures 4B and 4C). The downregulated “Carbon metabolism” term comprised mRNAs encoding enzymes of the glycolytic pathway (*Glut1*, *Hk1*, *Gck*, *Adpgk*, *Eno1b*, *Pfkl*, *Pfkp*, *Pgk1*, *Pgam1*, *Eno1*, *Eno2*, *Pdha1*, and *Pdhb*), the pentose phosphate pathway (*Tkt*, *Taldo1*, *G6pdx*), and the tricarboxylic acid (TCA) cycle (*Aco1*, *Aco2*, *Idh3a*, *Idh3g*, *Sucla2*, *Suclg1*, *Suclg2*, *Fh1*, and *Mdh2*) as well as the glutamate dehydrogenase *Glud1*, and the malic enzymes *Me1*, *Me2*, and *Me3* (Figures 4B and 4C). There was also a downregulation of the “Endocytosis” term, which comprised 54 mRNAs, almost all of them encoding regulators of clathrin-coated pit-dependent and clathrin-independent endocytosis, and of endosome recycling (Figure 4B).

Collectively (Figure 4D), these observations show that, in soleus muscle, increased glycemic levels and lower whole body insulin sensitivity were associated with reduced expression of mRNAs controlling insulin signaling, glycolysis, pentose phosphate pathway, TCA cycle, and OXPHOS activities as well as lower expression of mRNAs encoding regulators of endocytosis/exocytosis, which may reduce insulin-dependent trafficking of Glut4 to the plasma membrane and further decrease glucose metabolism.^16^^,^^52^ Notably, all these downregulated terms were also found when the analysis was performed only with the RC fed mouse data, suggesting a primordial role of the genetic background in determining the expression levels of these mRNAs and indicating that HFD feeding had relatively low influence on mRNA expression in muscle as compared their expression in the other tissues studied (Figures S4A and S4B).

#### Liver

**Figure 5:**
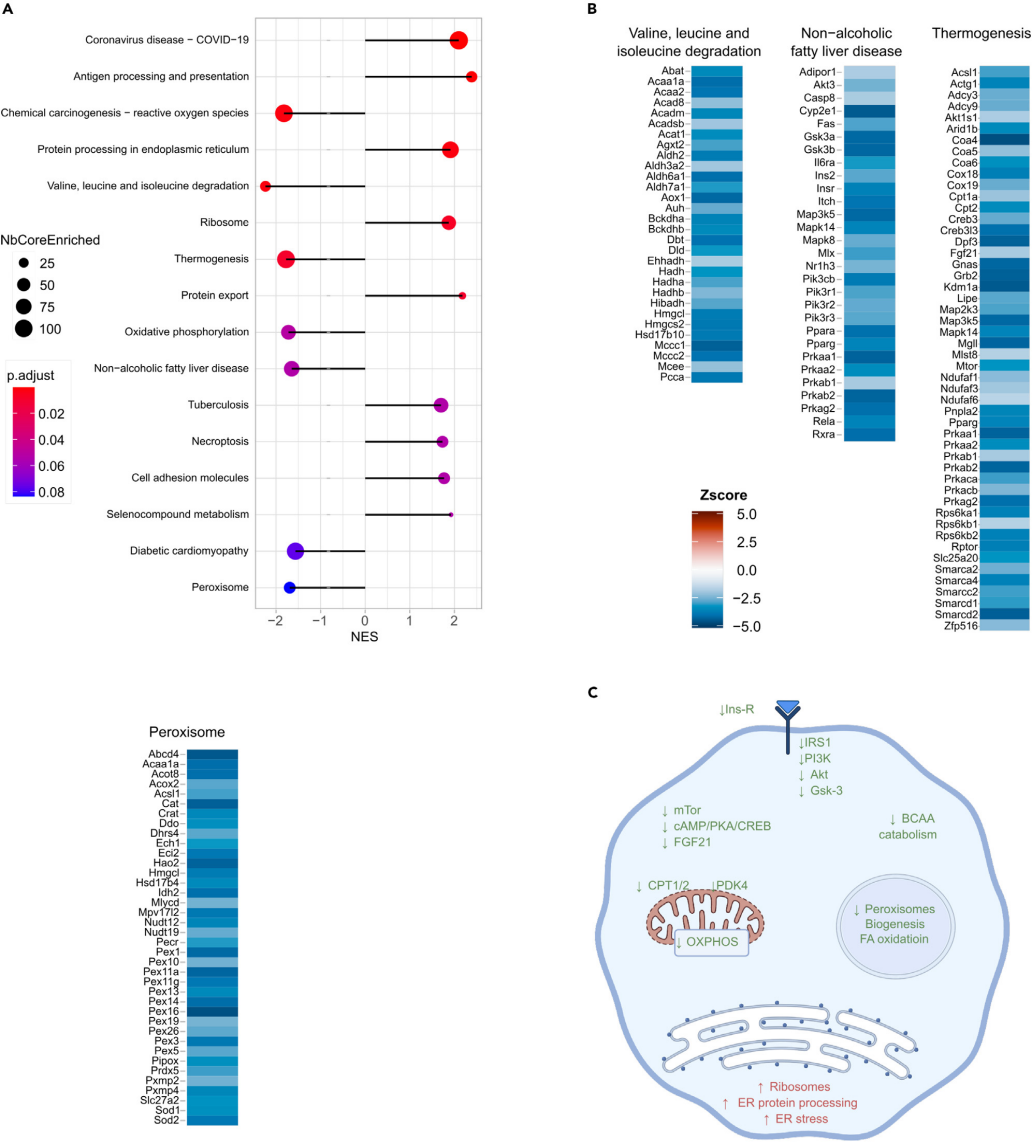
Pathways regulated with glycemia in liver (A) Plot showing the major KEGG pathways regulated with glycemia in liver. (B) Illustration of the mRNAs that comprise selected KEGG terms with the color indication of their *Z* score value. (C) Scheme of the major pathways down and upregulated with glycemia and that are involved in the control liver function.

Figure 5A shows that seven terms were downregulated and nine upregulated with the glycemic levels. The downregulated terms were categorized in four groups. The “Chemical carcinogenesis-reactive oxygen species” term contained a unique set of mRNAs, including seven glutathione-S-transferase genes (Table S4), suggesting decreased reactive oxygen species (ROS) scavenging capacity; as ROS reduce insulin signaling in liver,^53^ this observation is compatible with the inverse correlation between glycemia and whole body insulin sensitivity (Figure 1C). This term also contained mRNAs for various signaling kinases and phosphatases, including activators of the NFkB pathway (*Chuk*, *Prkd2*, and *Map3k14*). The “Valine, leucine, and isoleucine degradation” term included several mRNAs of the BCAA degradation pathway, in particular, *Bckdha* and *Bckdhb*, which encode subunits of the first and rate-limiting enzyme in BCAA degradation (Figure 5B). It has previously been documented that lower BCAA degradation increases circulating levels of BCAAs, which contribute to increased insulin resistance.^54^^,^^55^

The terms “Oxidative phosphorylation”, “Non-alcoholic fatty liver disease” and “Diabetic cardiomyopathy” contained mRNAs that were almost all represented in the “Thermogenesis” term. In this term, out of 48 mRNAs, 23 encode subunits of the OXPHOS chain or of proteins required for OXPHOS chain assembly (Figure 2B). Reduced OXPHOS activity is linked to insulin resistance in the liver.^18^ The other downregulated mRNAs in these terms were associated with the mTor pathway (*mTor*, *Rptor*), the cAMP pathway (*Gnas*, *Creb3l3*), AMP-activated protein kinase (*Prkaa1*, *Prkaa2*, *Prkab2*, and *Prkag2*), the insulin/tyrosine kinase signaling pathway (*Insr*, *Grb2*, *Pten*, *Mapk14*, *Mp3k5*, *Gsk3a*, and *Gsk3b*), and with mRNAs involved in fatty acid catabolism (*Ppara*, *Pparg*, *Rxra*, *Cpt2*, *Slc25a20*, and *Pnpla2*) (Figure 5B). The fourth down-regulated term, “Peroxisome”, included several mRNAs involved in peroxisome biogenesis (*Pex*s), fatty acid transport (*Slc27a2*), fatty acid oxidation (*Pecr*, *Hsd17b4*, *Ech1*, *Eci2*, and *Acot8*) and ROS degradation (*Cat*, *Sod1*, and *Sod2*) (Figure 5B). This aligns with the reduced expression of *Ppara*, *Pparg*, and *Rxra*, which are key transcription factors controlling peroxisome biogenesis.

The downregulation of these mRNAs identified insulin signaling, OXPHOS, BCAA degradation, fatty acid degradation, and ROS scavenging as the main pathways that could explain reduced hepatic and whole-body insulin sensitivity. Notably, these pathways were identified when the analysis was conducted with the combined RC and HFD data or only with the RC data indicating that their activity was predominantly determined by the mouse genetic background (Figures S5A and S5B).

Nine terms were upregulated. The “Coronavirus disease-COVID-19" term contained 58 mRNAs, of which 35 encode ribosomal proteins, which were also present in the “Ribosome” term (Table S4). The other mRNAs in this term encode several receptors for components of the innate immunity system (*Mbl2*, *Gcgr3*, *Tlr2*, *Tlr7*, *Cd74*, *Fcer1g*, *Fcgr1*, *Fcgr2b*, *Fcgr3*, *Fcgr4*, *Il10ra*, *Il10rb*, *Clec7a*, and *Mrc1*), related intracellular signaling protein (*Pik3c3*, *Plcg2*, and *Syk*), and the complement pathway (*C6*, *Cr1l1*, *C7*, *C8b*, and *C9*); many of these genes were also present in the “Tuberculosis” term (Table S3). As many of these genes are expressed by T lymphocytes or macrophages, they suggest increased immune cells infiltration in the liver with higher glycemic levels.

The “Antigen processing and presentation”, “Protein processing in endoplasmic reticulum”, “Protein export” and “Ribosome” terms included mRNAs encoding components of the protein biosynthesis pathways (the “Ribosome” genes) and of proteins required for the translocation of nascent proteins into the ER, for protein folding and quality control, for ER to Golgi transport, and for ERAD (Table S4). There was also higher expression of *Xbp1* and *Ern1*, the enzyme that controls *Xbp1* splicing and activation, indicating that increased protein biosynthesis activity was associated with higher unfolded protein response and ERAD activity, which both preserve ER homeostasis.^56^ These upregulated terms were found in the analysis of the combined RC and HFD mouse data but also when the analysis was carried out only with the HFD mouse data, suggesting that they are related to the metabolic stress induced by high calorie-containing food (Figures S6A and S6B).

Collectively, the pathways that were down-regulated with higher glycemic levels (insulin signaling, OXPHOS, BCAA degradation, fatty acid degradation in mitochondria and peroxisomes) point to insulin resistance in hepatocytes and BCAA-mediated whole-body insulin resistance (Figure 5C). The upregulated terms suggest increased liver infiltration by immune cells and a coordinated increase in the expression of mRNAs regulating protein biosynthesis, protein translocation into the ER, protein folding, ERAD, and protein transport from the ER to the Golgi. As in muscle, these observations suggest higher protein biosynthesis activity, although in muscle these processes were related to pre-translational control mechanisms (mRNA splicing, nucleocytoplasmic transport, and ribosome biogenesis) (Figure 4).

#### Visceral adipose tissue

**Figure 6:**
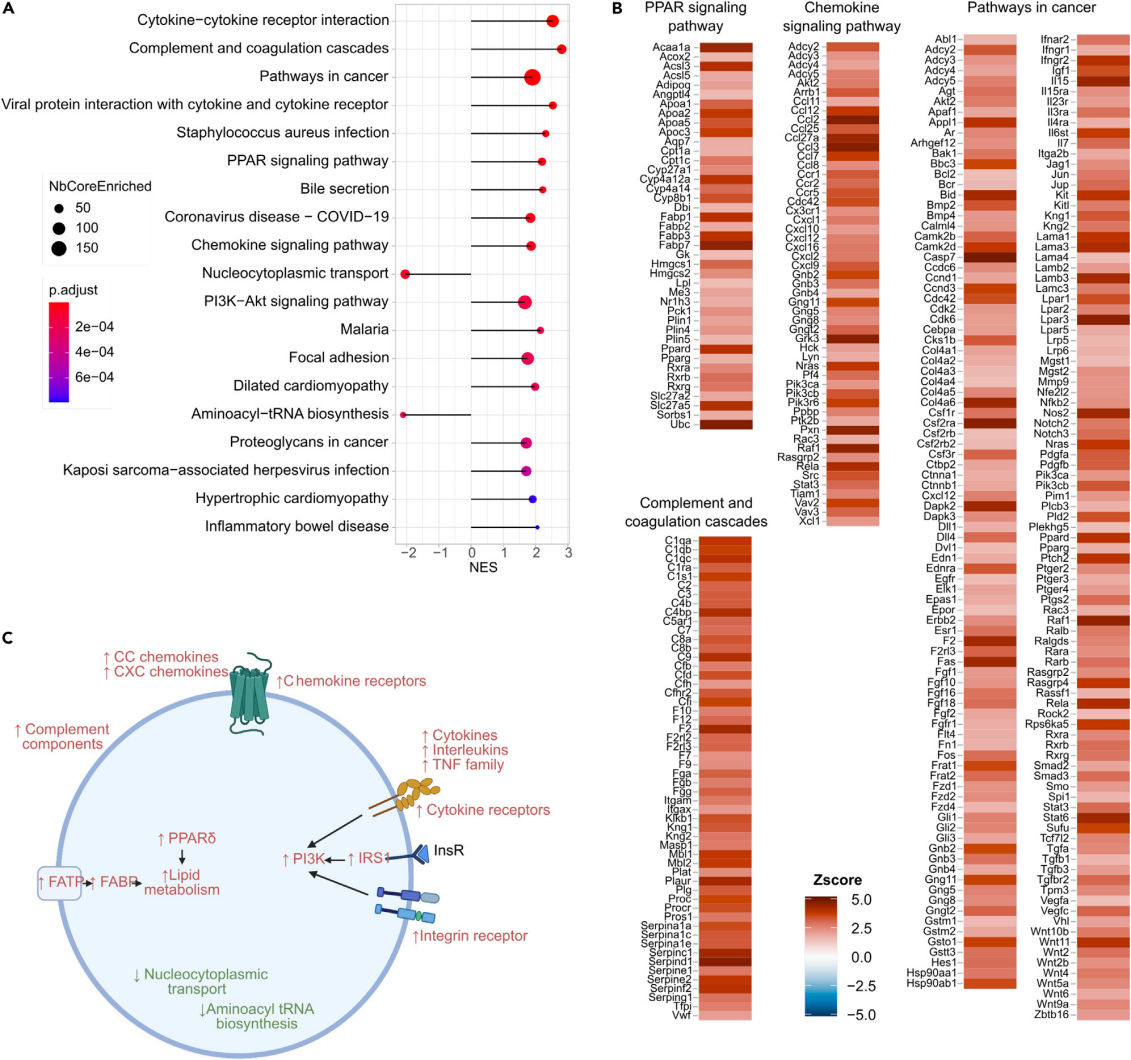
Pathways regulated with glycemia in visceral adipose tissue (A) Plot showing the major KEGG pathways regulated with glycemia in visceral adipose tissue. (B) Illustration of the mRNAs that comprise selected KEGG terms with the color indication of their *Z* score value. (C) Scheme of the major pathways down and upregulated with glycemia and that are involved in the control of adipose tissue function and inflammation.

In this tissue, the identified terms were mostly upregulated with glycemic levels (Figure 6A). The terms “Cytokine-cytokine receptor interaction”, “Viral protein interaction with cytokine and cytokine receptor”, “Chemokine signaling pathway”, “Pathways in cancer”, “Pi3k-Akt signaling pathway”, “Focal adhesion” showed striking, coordinated increased expression of a multitude of signaling pathways related to innate immunity (Figure 6B and Table S5). The upregulated mRNAs encoded CC chemokines (*Ccl*, *Ccl2*, *Ccl3*, *Ccl7*, *Ccl11*, and *Ccl25*), CXC chemokines (*Cxcl*, *Cxcl1*, *Cxcl2*, *Cxcl4*, *Cxcl9*, *Cxcl10*, *Cxcl12*, *Cxcl14*, and *Cxcl16*), and some of their receptors (*Ccr1*, *Ccr2*, *Ccr5*, *Cxccr*, and *Cx3cr1*). In addition, these terms also included mRNAs for interleukins (*Il-1*, *Il-17*, *Il-18*, and *Il-33*) and interleukin receptors (*Il10rb*, *Il6st*, *Il2rb*, and *Il22Ra1*) as well as the receptors for interferon and tumor necrosis factor (TNF) family receptors (*Ifnar2*, *Ifngr2*, *Tnfr1*, *Tnfr2*, *Ltbr*, *Fas*, *Dr4*, and *Dr5*) (Figure 6B). The “Pathways in cancer” term (Figure 6B) also included several mRNAs for the *Bmp*, *Dll*, *Fzd*, and *Vegf* families of ligands, for various receptors (*Csfr2a*, *Csf3r*, *Ednra*, *Ifngr2*, *Notch*, *Ptch*, *Tgfbr2*, and *Lpar3*), and for intracellular signaling molecules (*Stat3*, *Stat6*, *Gnb2*, *Gng11*, *Gngt2*, *Camkd2*, *Pi3kcb*, and *Rela*), which are all involved in cellular differentiation and function. It also included mRNAs encoding ECM proteins (*Lams*, *Col4as*); interactions of pre-adipocytes and adipocytes with the extracellular matrix are also crucial to support adipogenesis and to maintain the function of differentiated cells.^57^

Interestingly, the upregulated “Complement and coagulation cascade” (Figure 6B) and “Staphyloccocus aureus infection” terms included many mRNAs encoding components of the complement cascade (Figure 6B). Complement proteins are produced by adipocytes and their secretion and activation of the complement cascade favor the development of insulin resistance and fibrosis.^58^^,^^59^ Finally, the upregulated “Ppar signaling pathway” (Figure 6B) included *Pparb*/*d*, *Rxr*, the fatty acid transporters (*Slc27a5*), the fatty acid binding proteins *Fabp1*, *Fabp3*, *Fabp7* and genes involved in fatty acid metabolism (*Cpt1*, *Acsl3*, *Hmgcs1*, *Acaa1a*, *Cyp4a1*, and *ThiolaseB*), suggesting increased fatty acid uptake and metabolism capacity.

Only two KEGG terms were down-regulated, the “Nucleocytoplasmic transport” and the “Aminoacyl-tRNA biosynthesis” terms (Table S5). The first one included 15 mRNAs encoding nuclear pore complex subunits and proteins regulating protein nuclear import and export. A few of these mRNAs (*Dxd19b*, *Kpnb1*, *Xpo5*, and *Upf2*) were also regulated in muscles, but in an opposite direction. The other terms included 12 mRNAs for cytosolic and mitochondrial aminoacyl tRNA synthases. The downregulation of these pathways suggests reduced protein translation activity.

Collectively, the previous observations indicate that higher basal glycemic levels were associated with increased adipose tissue inflammation and increased activity of multiple innate immunity pathways. At least some of these inflammatory/innate immunity components can be produced by adipocytes (complement proteins, *Il-6*, *TNFa*, *Il-15*, *Il-33*, *Il34*, and *CCL2*) whereas others (chemokines, interleukins) are produced by monocytes/macrophages, endothelial cells, fibroblasts and other recruited inflammatory cells. A consequence of this inflammatory state is the development of insulin resistance in adipocytes.^60^ Although the insulin signaling components *InsR*, *Irs1*, *Pi3K*, and *Akt* were upregulated, the massive upregulation of inflammatory mRNAs may induce insulin resistance by posttranslational Ser/Thr phosphorylation of the Insr and Irs1. Notably, no genes involved in glucose uptake (Glut4), glycolysis, TCA cycle, or OXPHOS were found related to basal glycemia, further supporting the role of inflammation and interaction with the extracellular matrix as physiological regulators of insulin sensitivity in adipocytes. Finally, these pathways were identified when the analysis was restricted to the data from the RC mice, from the HFD mice, or from the combined analysis of both, suggesting combined effect of the genetic background and the metabolic stress on mRNAs expression (Figures S6A and S6B).

### Lipids

**Figure 7:**
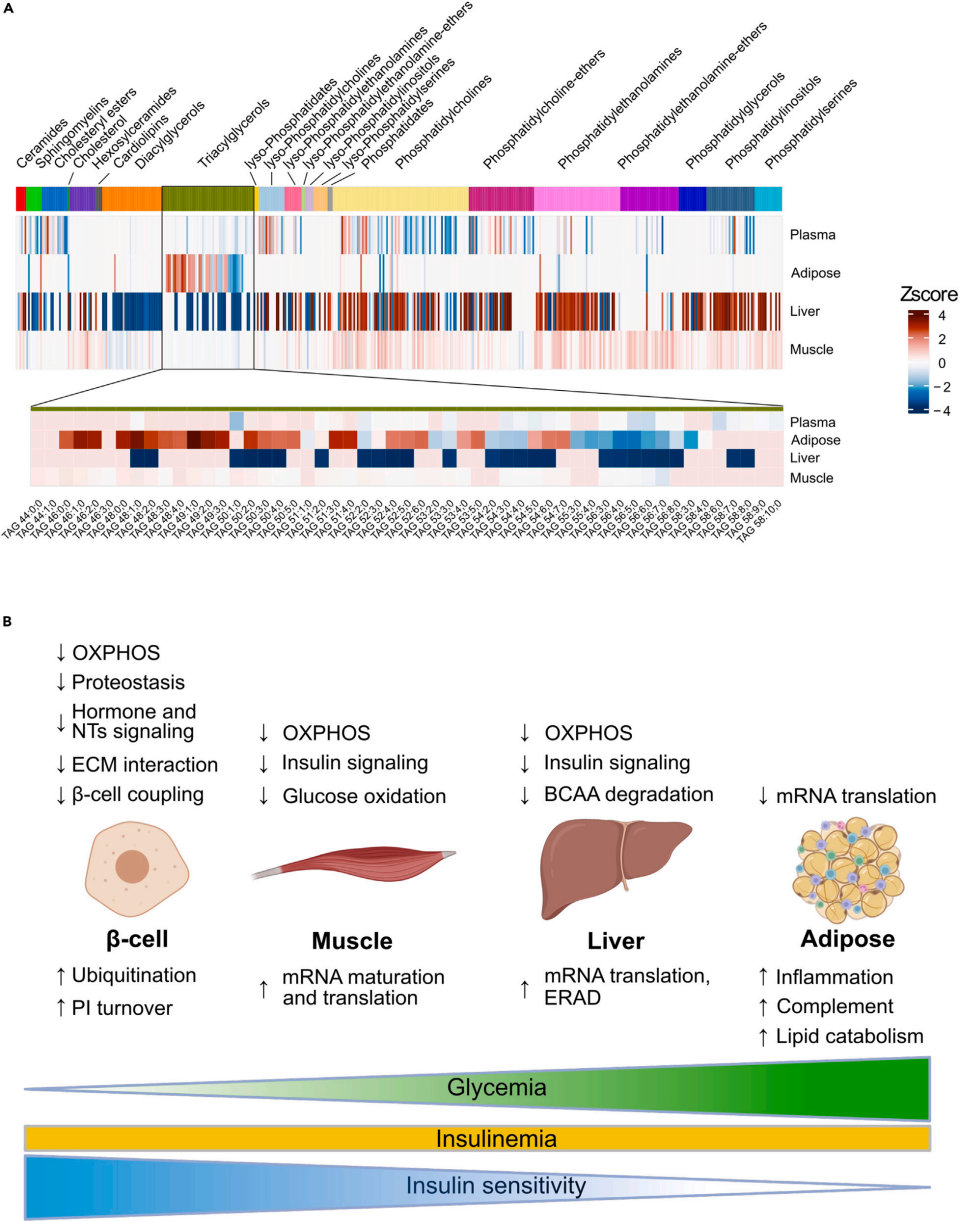
Lipidomic data and summary scheme (A) *Top*: heatmap of the lipid species that are up or downregulated with glycemia in the indicated tissues. *Bottom*: heatmap displaying the structure of the individual TAGs in each organ. Relative abundance is expressed as Z-scores. (B) Summary of the tissue-specific changes in signaling and metabolic pathways that are coordinately regulated with fasting glycemia (for discussion see text).

Up to 338 lipids, belonging to several classes (diacylglycerols (DAGs), triacylglycerols (TAGs), phospholipids and lysophospholipids, sphingomyelins, ceramides, and cholesterol) were measured in liver, adipose, muscle, and plasma (Figures 1E and 7A, and Table S6). Their molar amounts related to the glycemic levels are presented for each tissue in the heatmap of Figure 7A. The largest number of lipids regulated with glycemia were found in the liver with a downregulation of DAGs and TAGs with fatty acids of different lengths and desaturation levels (Figure 7A) and an upregulation of phosphatidylcholines (PCs), phosphatidylethanolamines (PEs) (with the structure PE 18:0_22:4, 18:0_22:5; 18:0_22:6), and phosphatidylinositols (PIs) (Table S6 for all lipidomic data). In muscle, most phospholipids were upregulated, but only to a very moderate level. In adipose tissue, low molecular weight TAGs (TAG 46:1; 0 to TAG 50:3; 0) were upregulated whereas larger TAGs with higher levels of desaturation (TAG 56:3; 0 to TAG 56:8; 0) were downregulated (Figure 7A). In plasma there was a complex pattern of lipids up and downregulated with glycemia, with a notable decreased concentration of several PCs and PIs and a marked increase in Cer 42:2; 2 and of some lysoPCs (Table S6). Thus, reduced levels of DAGs and TAGs in the liver and increased short chain fatty acid-containing TAGs of the visceral fat were the most associated with basal glycemia.

### Conclusion

The present transcriptomic and lipidomic data fusion approach allowed to identify pathways that are coordinately regulated in pancreatic islets, soleus muscle, liver, and visceral adipose tissue with fasting glycemia. Although, we identified these pathways based on transcriptomic data, extrapolation from mRNA expression to pathway activity is likely to be mostly correct. Indeed, studies have demonstrated that when the expression of mRNAs pertaining to a given biological pathway are coordinately up or downregulated, this reflects a congruent change in the activity of this pathway.^61^ Similarly, when an mRNA encoding an unknown protein is coregulated with mRNAs belonging to a defined pathway, the unknown protein usually contributes to the activity of such pathway, as reported in previous studies.^62^^,^^63^^,^^64^^,^^65^^,^^66^ Thus, analysis of transcriptomic datasets can be highly informative on tissue-specific physiological functions and their regulations.

Several striking features emerged from our study (Figure 7B). First, in islets, constant insulinemia despite higher glycemic levels was associated with decreased expression of several pathways that normally potentiate GSIS. These include receptors that inform the β cells about the metabolic status of peripheral organs and the central nervous system and about local ECM environment, and gap junction proteins that support electrophysiological coupling of β cells. Importantly, the main components of the Glut2-Gck-K_ATP_ channel signaling pathway that controls GSIS were not regulated with glycemic levels. This emphasizes the role of the β cell as an integrator of a multitude of metabolic, hormonal, and immune cues that modulate insulin secretion in response to changes in peripheral organ metabolic status. Defects in any axis of this intricate β cell interorgan communication system may, thus, deregulate insulin secretion and potentially lead to the hyperglycemia that defines T2D. A more complete understanding of these regulatory axis in health and T2D is warranted.

Signs of insulin resistance were observed in muscle, liver, and adipose tissue, however, the pathways involved were distinct in each tissue. In muscle, our analysis suggested reduced insulin signaling, lower glycolysis, TCA cycle, and OXPHOS activities, as well as reduced vesicular trafficking, possibly decreasing insulin-stimulated, Glut4-dependent glucose uptake. In liver, there was a downregulation of mRNAs encoding components of the insulin signaling cascade, of the OXPHOS chain, of anti-oxidant proteins and of key BCAA degradation enzymes, all potentially reducing insulin sensitivity. We also observed signs of increased activity of the immune system, which may also negatively impact hepatic insulin sensitivity. At the same time, however, mRNAs controlling the cAMP pathway were decreased, suggesting that insulin resistance was not associated with increased hepatic glucose production. In adipose tissue, decreased insulin sensitivity was associated with a massive increase in mRNAs for inflammatory proteins and their receptors and for proteins of the complement system. We also observed increased expression of integrins, which link adipocytes to the extracellular matrix; these interactions not only maintain the differentiated functions of adipocytes but also support adipogenesis. It is well known that inflammation can induce adipose tissue insulin resistance in obesity and diabetes.^67^^,^^68^^,^^69^^,^^70^ However, the mice we studied were neither obese nor diabetic, thus our observations suggest that adipose tissue inflammation is a physiological mechanism that fine-tunes insulin sensitivity, and possibly adipogenesis in response to changes in glycemic levels.

In conclusion, our study shows how a multitude of pathways are coordinately regulated across tissues to control fasting glycemia. This control is genetically determined and modulated by the diet and by the interaction between both factors. This study also indicates that although glucose homeostasis can be described as the results of the equilibrium between GSIS and insulin action, a complete description of the control of glucose homeostasis needs to integrate the interactions of a multitude of pathways that control the secretion of various hormones, their action on several tissues, as well as local and systemic metabolic, inflammatory, and neuronal processes. How this system is globally controlled is starting to be understood. However, because the overarching aim of the system is to control blood glucose concentrations, glucose itself may play a cardinal regulatory role by controlling transcriptional activity in the tissues investigated here (for instance, through *Chrebp*), by triggering hormone secretion, by controlling immune cell function, or the activity of the autonomous nervous system. In this context, our study provides a resource to help guide future studies of interorgan communications in the control of glucose homeostasis.

### Limitations of the study

A limitation of our conclusions on the pathways coordinately regulated with glycemia is that they are based on the analysis of the transcriptome of the selected tissues. Changes in mRNA expression may not always be associated with a congruent change in protein expression. Conversely, protein expression may also be regulated at the translational level without changes in the abundance if their cognate mRNAs; such occurrence could not be detected by transcriptomic analysis. Also, inclusion of the transcriptome of other tissues involved in glucose handling and sensing, such as intestine, kidney, or brain are not included in our analysis nor is the gut microbiota, which may all influence basal glycemia. Finally, our study included only male mice and, thus, cannot identify potential sex-specific differences.

## Resource availability

### Lead contact

Requests for further information and resources should be directed to and will be fulfilled by the lead contact, Bernard Thorens, Center for Integrative Genomics, University of Lausanne and Swiss Institute for Bioinformatics, Lausanne, Switzerland; e-mail: bernard.thorens@unil.ch.

### Materials availability

This study did not generate new unique reagents.

### Data and code availability

• Data availability

Transcriptomic data for muscle, liver and adipose tissue have been deposited in NCBI’s Gene Expression Omnibus with the accession number GSE164672 (https://www.ncbi.nlm.nih.gov/geo/query/acc.cgi?acc=GSE164672).^30^ Pancreatic islets transcriptomic data are available with the accession number GSE140369 (https://www.ncbi.nlm.nih.gov/geo/query/acc.cgi?acc1/4GSE140369).^30^

Lipidomics data were deposited in Zenodo with the accession number https://doi.org/10.5281/zenodo.13827925.

Any additional information required to reanalyze the data reported in this work is available from the lead contact upon request.

• Code availability

This paper does not report original code.

• All other items

There are no other items.

## Acknowledgments

This project has received funding from the 10.13039/501100010767Innovative Medicines Initiative 2 Joint Undertaking under grant agreement no 115881 (RHAPSODY). This Joint Undertaking receives support from the European Union’s Horizon 2020 research and innovation program and EFPIA. This work is also supported by the Swiss State Secretariat for Education, Research and Innovation (SERI) under contract number 16.0097. The opinions expressed and arguments employed herein do not necessarily reflect the official views of these funding bodies. B.T. also received support from a 10.13039/501100001711Swiss National Science Foundation grant (310030_1824969).

## Author contributions

F.M. performed data curation, analysis, and figure preparation; A.R.S.A., M.P., and I.M. performed data analysis; M.G., C.K., and K.S. performed lipidomic analysis; C.C.G., H.L.S., K.M., J.L., and J.D.: performed mouse physiological experiments and data analysis; C.M., M.I., and B.T.: conceptualized the work, acquired funding, interpreted the data and wrote the original draft; all authors revised and edited the manuscript.

## Declaration of interests

M.G., C.K., and K.S. are employees of Lipotype GmbH.

## STAR★Methods

### Key resources table

**Table:** 

REAGENT or RESOURCE	SOURCE	IDENTIFIER
**Critical commercial assays**		

Insulin RIA/ELISA	Crystal Chem Inc.	Cat#90080
Glucose monitoring system	A. Menarini Diagnostics, France	Glucofix Tech

**Deposited data**		

Transcriptomic data muscle, liver and adipose tissue	Sánchez-Archidona et al.^30^	GEO: GSE164672
Pancreatic islets transcriptomic data	Sánchez-Archidona et al.^30^	GEO: GSE140369
Lipidomics data	This paper	https://doi.org/10.5281/zenodo.13827925

**Experimental models: Organisms/strains**		

Mice C57Bl/6, DAB2, Balb/c	Janvier-Labs	Cat#C57BL/6JRj, Cat#DBA/2JRj, Cat#BALB/cJRj

**Software and algorithm**s		

MATLAB 9	The MathWorks, Inc.	https://ch.mathworks.com/fr/products/matlab.html
R	R Foundation for Statistical Computing, Vienna, Austria.^71^	https://www.r-project.org/
STAR-2.5.3a	Dobin. et al.^72^	https://github.com/alexdobin/STAR
edgeR	Robinson et al.^73^	https://bioconductor.org/packages/release/bioc/html/edgeR.html
Limma	Ritchie et al.^74^	https://bioconductor.org/packages/release/bioc/html/limma.html
missForest	Stekhoven et al.^75^	https://cran.r-project.org/web/packages/missForest/index.html
WGCNA	Langfelder and Horvath^31^	https://cran.r-project.org/web/packages/WGCNA/index.html
consensusOPLS	Boccard et al.^33^	https://gitlab.unige.ch/Julien.Boccard/consensusopls
KOPLS-DA	Rantalainen et al.^76^ and Bylesjo et al.^77^	https://kopls.sourceforge.net/index.shtml
clusterProfiler	Wu et al.^78^ and Yu et al.^79^	https://bioconductor.org/packages/release/bioc/html/clusterProfiler.html
LipidXplorer	Herzog et al.^80^^,^^81^	https://lifs-tools.org/lipidxplorer.html

**Other**		

Regular chow	SAFE (Route De Saint Bris, 89290 AUGY, France)	Cat#SAFEA04
High fat, high sucrose diet	SAFE (Route De Saint Bris, 89290 AUGY, France)	Cat#SAFE235F

### Experimental model and study participant details

Eight weeks old male C57Bl/6J, DBA/2J and BALB/cJ mice were used. They were maintained in a 12 hours/12 hours light /dark cycle and had *ad libitum* access to either a high fat, high sucrose diet (SAFE 235F, with 46% fat expressed in Kcal/kg) or a regular diet (SAFE A04). Mice were euthanized after a 5-hour fast. Institutional permission was obtained from Buffon Animal Facility agreement: N° B 75-13-17.

Ethical authorization number granted by French Ministry of Research : 201601261121896.

### Method details

#### Physiological and biochemical analysis

Insulin tolerance tests (ITT, Novorapid, 0.5UI/kg) were performed in five hours fasted mice on days 2, 10 and 30 (ref). Glycemia were measured using a glucometer (A. Menarini Diagnostics, France), and insulin resistance was calculated as the area under the curve of glycemia (AUC; mg/dL∗t) measured at 0, 15, 30, 45, 60, 90 and 120 minutes after insulin administration. Basal (five hours fasted) insulinemia were measured using an Ultra-Sensitive Mouse Insulin ELISA Kit (Crystal Chem Inc., #90080). The number of mice used in these phenotyping experiments ranged between 185 and 195.

#### RNAseq analysis

cDNA libraries were prepared from RNA isolated from mouse tissues using Illumina TruSeq protocol. RNA-Seq was performed on the Illumina HiSeq platform to generate ∼40Mio 125nt single-end reads per sample. Reads were mapped and quantified with STAR-2.5.3a software^72^ using M.musculus-mm10 as reference genome and GRCm38.83 from ENSEMBL as the reference annotation index. For each sample, quality control included verification of the total number of reads, percent of uniquely mapped reads, number of detected expressed genes, gene body coverage and cumulative gene diversity. The resulting counts per gene from different samples were integrated to construct a single count matrix for each tissue that was filtered, excluding those genes with less than one count per million with 'edgeR'.^73^ We excluded three clear outliers identified by principal component analysis (PCA) and hierarchical clustering in the islets data set. The count matrix was normalized using trimmed mean (TMM) normalization method. Differentially expressed genes comparing HF and RC, and the different strains were detected using the limma package in R.^71^^,^^74^ P-values were adjusted for multiple comparisons with the Benjamini Hochberg procedure,^82^ and those genes whose adjusted pvalue≤0.05 were considered as differentially expressed.

#### Weighted Gene Correlation Network Analysis (WGCNA)

WGCNA was performed on the RNA-Seq data from all time points, mouse strains and diets to generate modules of co-expressed genes.^31^ Co-expression networks for each tissue were constructed by calculating signed adjacency matrices using a soft-thresholding power of 6 and a pair-wise Pearson correlation between all genes. A signed topological overlap matrix (TOM) was then calculated from each adjacency matrix, converted to distances, and clustered by hierarchical clustering using average linkage clustering. Modules were identified in the resulting dendrogram by the Dynamic Hybrid tree cut with a cut height of 0.995 and a minimum module size of 20 genes. A PCA was calculated for each module in each data set using only module constituent genes to obtain the summarized values of expression of each module (the first principal component or eigenvalues). Because islets data and data from other tissues and plasma were acquired from different mice, 18 mice groups were defined by the three strains, two diets and three time points of harvesting. Module eigenvalues were summarized per mouse group using the mean.

#### Lipidomics

Visceral adipose tissue, liver, soleus muscle and plasma lipids were measured by mass spectrometry at the Lipotype shotgun lipidomics platform. Samples processing, lipid extraction, spectra acquisition and data processing and normalization were as described in Surma et al. 2015.^83^ The internal standard mixture contained: cholesterol D6 (chol), cholesterol ester 20:0 (CE), ceramide 18:1;2/17:0 (Cer), diacylglycerol 17:0/17:0 (DAG), phosphatidylcholine 17:0/17:0 (PC), phosphatidylethanolamine 17:0/17:0 (PE), lysophosphatidylcholine 12:0, (LPC) lysophosphatidylethanolamine 17:1 (LPE), triacylglycerol 17:0/17:0/17:0 (TAG) and sphingomyelin 18:1;2/12:0 (SM). Samples were analyzed by direct infusion in a QExactive mass spectrometer (Thermo Scientific) in a single acquisition. Tandem mass-spectrometry (MS/MS) was triggered by an inclusion list encompassing corresponding MS mass ranges scanned in 1 Da increments. MS and MS/MS data were combined to monitor CE, DAG and TAG ions as ammonium adducts; PC, PC O-, as acetate adducts; and PE, PE O- and PI as deprotonated anions. MS only was used to monitor LPE as deprotonated anion; Cer, SM and LPC as acetate adducts and cholesterol as ammonium adduct.

Lipidomic data were analyzed using LipotypeXplorer, a proprietary software developed by Lipotype GmbH, which is based on LipidXplorer.^80^^,^^81^ Only lipid identifications with a signal-to-noise ratio >5 and a signal intensity 5-fold higher than in corresponding blank samples were considered for further analysis. The median coefficient of lipid subspecies variation (RSD), as accessed by the repeated analysis of reference samples, was 7.5%.

Lipid species with ≥25% missing values across all available plasma samples were removed from the data set. For the lipids that remained in the data sets, missing values were imputed using a random forest approach, applying the function missForest from the R package missForest,^75^ with default parameters. Data were then normalized to the total signal (data = data / rowsums(data) ∗100). Data were not log transformed or further normalized. As for transcriptomics data, a WGCNA was run using signed network, Pearson correlation, soft thresholding power of 20 was used for plasma, liver and muscle, soft power of 12 was used for adipose, minimum module size of 5 for all tissues.^31^ To be consistent with transcriptomics data, 18 mice groups were defined by the three strains, two diets and three time points of harvesting. Module eigenvalues were summarized per mouse group using the mean.

### Quantification and statistical analysis

Pooled data of Figures 1B–1D are expressed as mean ± SEM; n represents the number of mice for each point as mentioned in the legend to the figure.

#### Multivariate statistical modelling

Common components and specific weights analysis (CCSWA) was performed using the eight tables of module eigengenes obtained from WGCNA on the lipidomics (Pearson correlation, soft thresholding power of 20 for plasma, liver and muscle, soft power of 12 used for adipose, minimum module size of 5 for all tissues) and transcriptomics (Pearson correlation, soft thresholding power of 6, minimum module size of 20 genes) datasets from different. Scatter plots visualizing sample distribution in the first few dimensions were produced using ggplot2 (version 3.5.0) in R.

Consensus Orthogonal Partial Least Squares (OPLS) analysis was performed using the MATLAB 9 environment. Consensus OPLS modelling was performed with the publicly available RVConsensusOPLS function (https://gitlab.unige.ch/Julien.Boccard/consensusopls, where modified RV-coefficients were computed with the publicly available MATLAB m-file^33^ and KOPLS-DA was assessed with routines implemented in the KOPLS open-source package^76^^,^^77^) on the same input data as CCSWA with 1 predictive latent variable and a maximum of 3 orthogonal variable, a 14 fold cross validation. Model significance was assessed by permutation (N=999) and the Q2 value used as a measure of model significance.

#### Pathway enrichment analysis

A functional enrichment analysis by Gene Set Enrichment Analysis (GSEA) was performed by tissue on unfiltered ranked gene lists. These ranked gene lists were obtained computing a Zscore for each gene as follows:

1. Compute $V_{i,k}=\sum_{j}{MM}_{ij}\cdot {sVIP}_{jk}$ where

*k* is the tissue

${MM}_{ij}$ is the module membership of the gene *i* to the module *j* obtained by ${MM}_{ij}=cor\left(x_{i\;},E_{j}\right)$ where $x_{i}$ is the expression of the gene *i* and $E_{j}$ is the module eigengene of the module *j.*

${sVIP}_{jk}$ is the signed Variable Importance in Projection of the module *j* of the tissue *k* k${sVIP}_{jk}={VIP}_{jk}\times sign\left({loadings}_{j}\right)$.

2. Compute ${Vperm}_{i,k}=\sum_{j}{MM}_{ij}\cdot {sVIPperm}_{jk}$ where ${sVIP}_{jk}$ is obtained from permuted Consensus OPLS models.
3. The Z-score is computed as follows ${Zscore}_{i,k}=\frac{V_{i,k}-\bar{{sVIPperm}_{jk}}}{{SD}_{{sVIPperm}_{jk}}}$

Genes are ranked by decreasing Z-score and the GSEA analysis is performed with the clusterProfiler gseKEGG function (R package version 4.6.2^78^^,^^79^ using Kyoto Encyclopedia of Genes and Genomes (KEGG) pathway collections for Mus Musculus. The enrichment scores were normalized by gene set size and their statistical significance was assessed by permutation tests (n=1,000). The list of all enriched terms across all tissues was restricted to the terms enriched with an adjusted p-value ≤ 0.01 in at least one tissue 48 terms.

### Additional resources

There are no additional resources.

## Supplemental information

Document S1. Figures S1–S6

Table S1. Samples metadata, related to Figure 1

Table S2. List of KEGG pathways and their mRNAs that are up- or down-regulated with glycemia in pancreatic islets, related to Figure 3

Table S3. List of KEGG pathways and their mRNAs that are up- or down-regulated with glycemia in soleus muscle, related to Figure 4

Table S4. List of KEGG pathways and their mRNAs that are up- or down-regulated with glycemia liver

Table S5. List of KEGG pathways and their mRNAs that are up- or down-regulated with glycemia in visceral adipose tissue, related to Figure 6

Table S6. List of lipids expressed in each organ and their Z-score values, related to Figure 7A
