# Supplementary material for: A multiorgan map of metabolic, signaling, and inflammatory pathways that coordinately control fasting glycemia in mice
Source: iScience. 2024 Oct 11;27(11):111134. doi: 10.1016/j.isci.2024.111134 (PMC11539597; doi:10.1016/j.isci.2024.111134)
Supplement: Document S1. Figures S1–S6 [file mmc1.pdf]

iScience, Volume *27*

Supplemental information

A multiorgan map of metabolic, signaling, and inflammatory pathways that coordinately control fasting glycemia in mice

Florence Mehl, Ana Rodríguez Sánchez-Archidona, Ida Meitil, Mathias Gerl, Céline Cruciani-Guglielmacci, Leonore Wigger, Hervé Le Stunff, Kelly Meneyrol, Justine Lallement, Jessica Denom, Christian Klose, Kai Simons, Marco Pagni, Christophe Magnan, Mark Ibberson, and Bernard Thorens

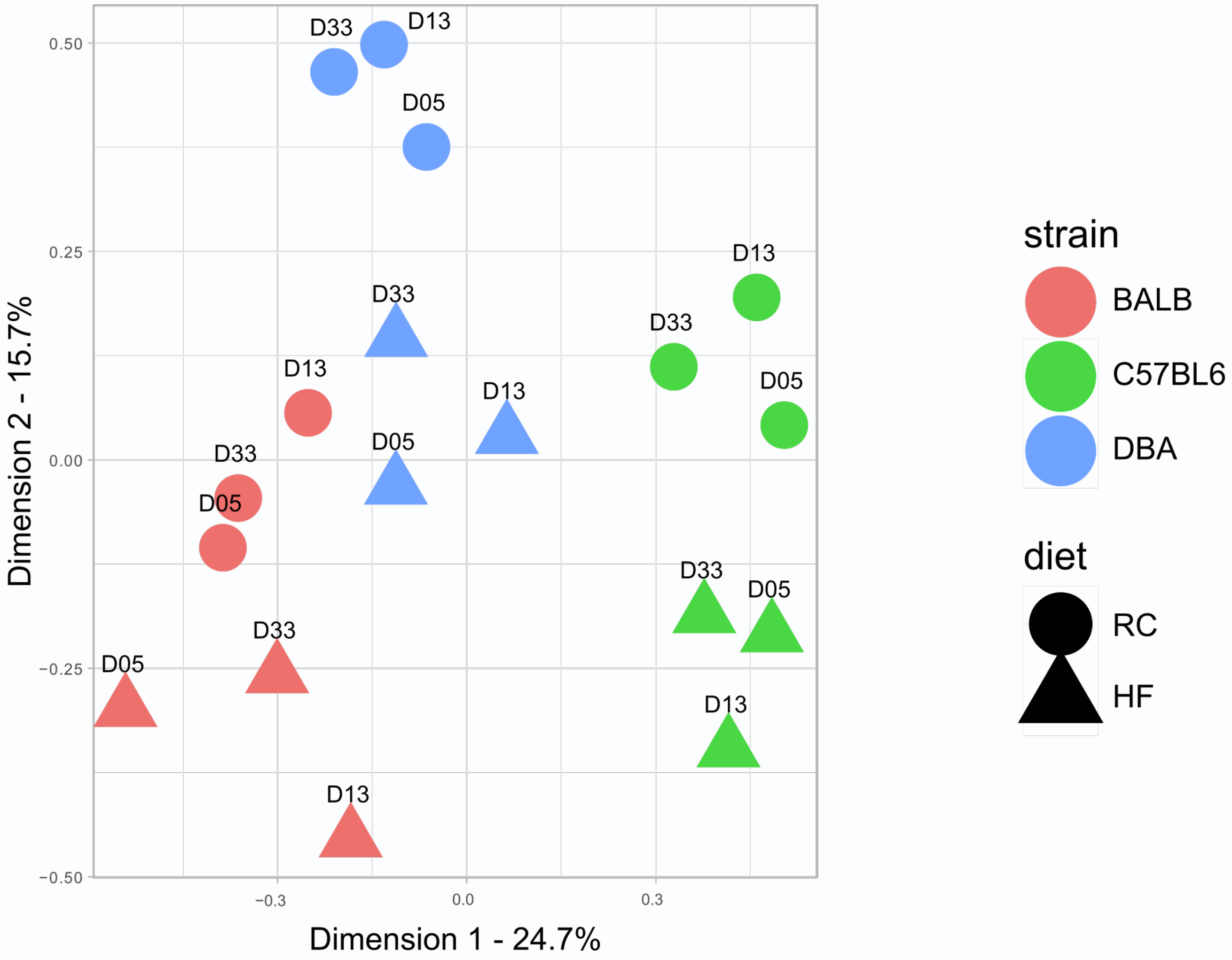

Figure S1. Scatterplot projecting the mice onto the two most important dimensions from the unsupervised multiblock analysis integrating transcriptomics and lipidomics data, related to Figure 1. The two dimensions represent >40% of the total observed variation (indicated on the axes). The representation is useful for determining similarities and differences between samples based on the integrated –omics data and demonstrates that the main source of variation is the strain (as indicated by separate clustering), followed by diet, which separates HF from RC fed mice along dimension 2.

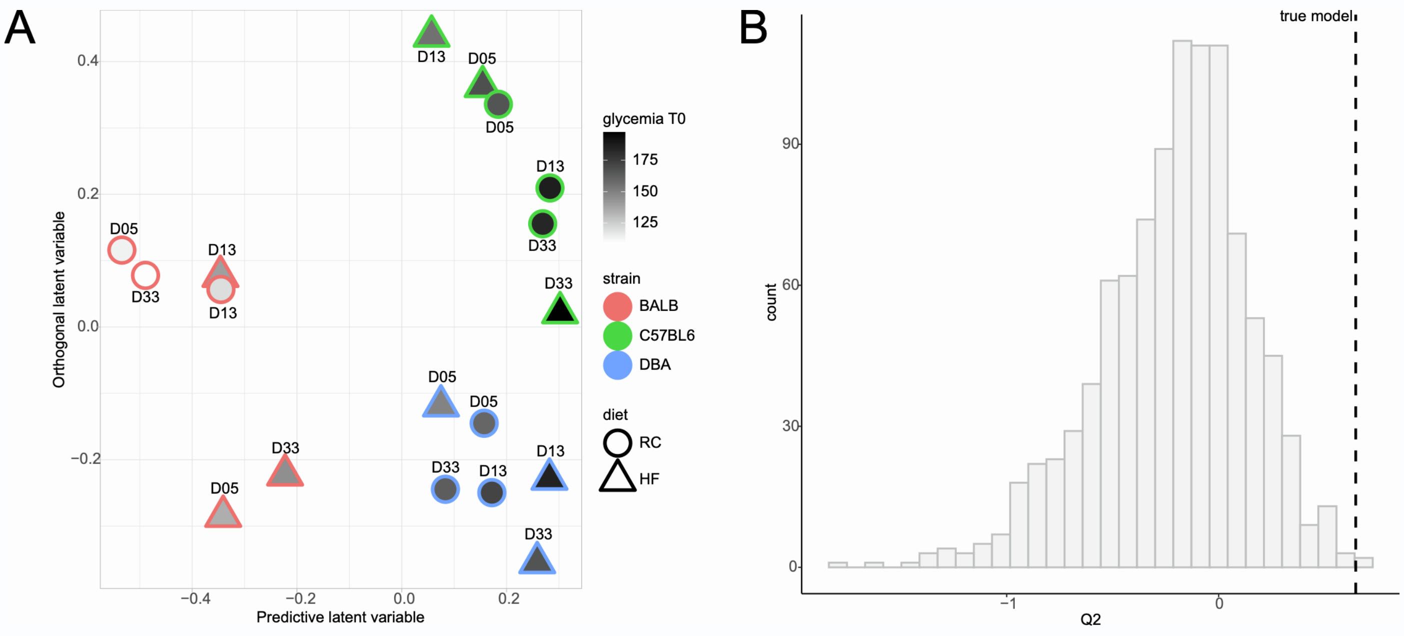

Figure S2. Supervised multiblock analysis integrating transcriptomics and lipidomics data, related to Figure 1. A.) Scatterplot showing projection of mouse samples onto the predictive and the first orthogonal components of a supervised multiblock model using basal glycemia as the predicted outcome. The plot shows that samples with differences in basal glycemia are distributed along the x axis representing the predictive component. B.) Histogram of Q^2 values (an estimate of the predictive ability of the model) obtained from 999 permuted models compared to the actual Q^2 of the model (dotted line) showing that the true model's predictive performance is significantly higher than the mean of the permuted models.

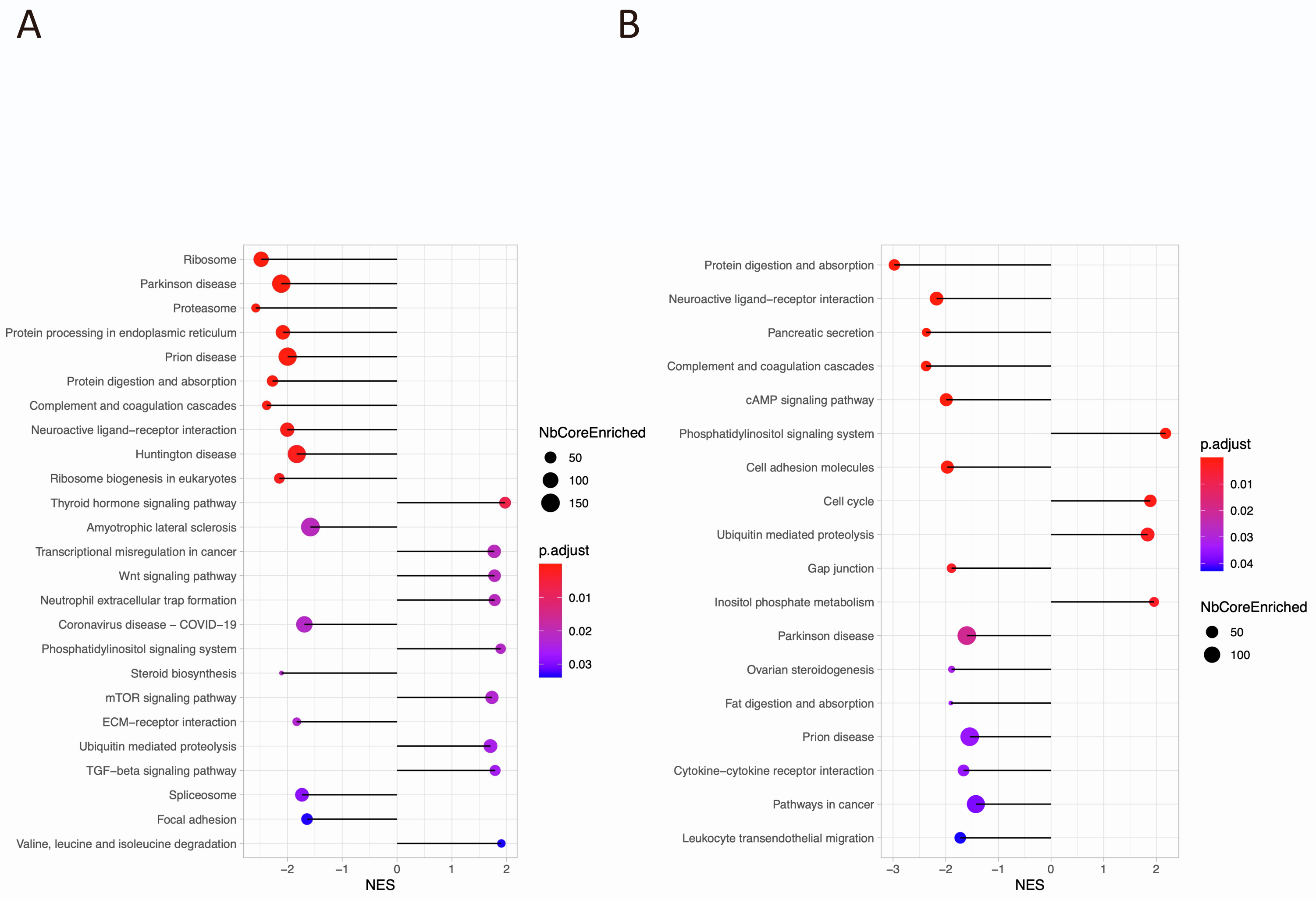

Figure S3: Islet KEGG pathways regulated with glycemia in RC or HFD fed mice, related to Figure 3. A.) Plot showing the major KEGG pathways regulated with glycemia (adjusted p ≤ 0.05) in islets from RC fed mice. B.) Plot showing the major KEGG pathways regulated with glycemia (adjusted p ≤ 0.05) in islets from HFD fed mice

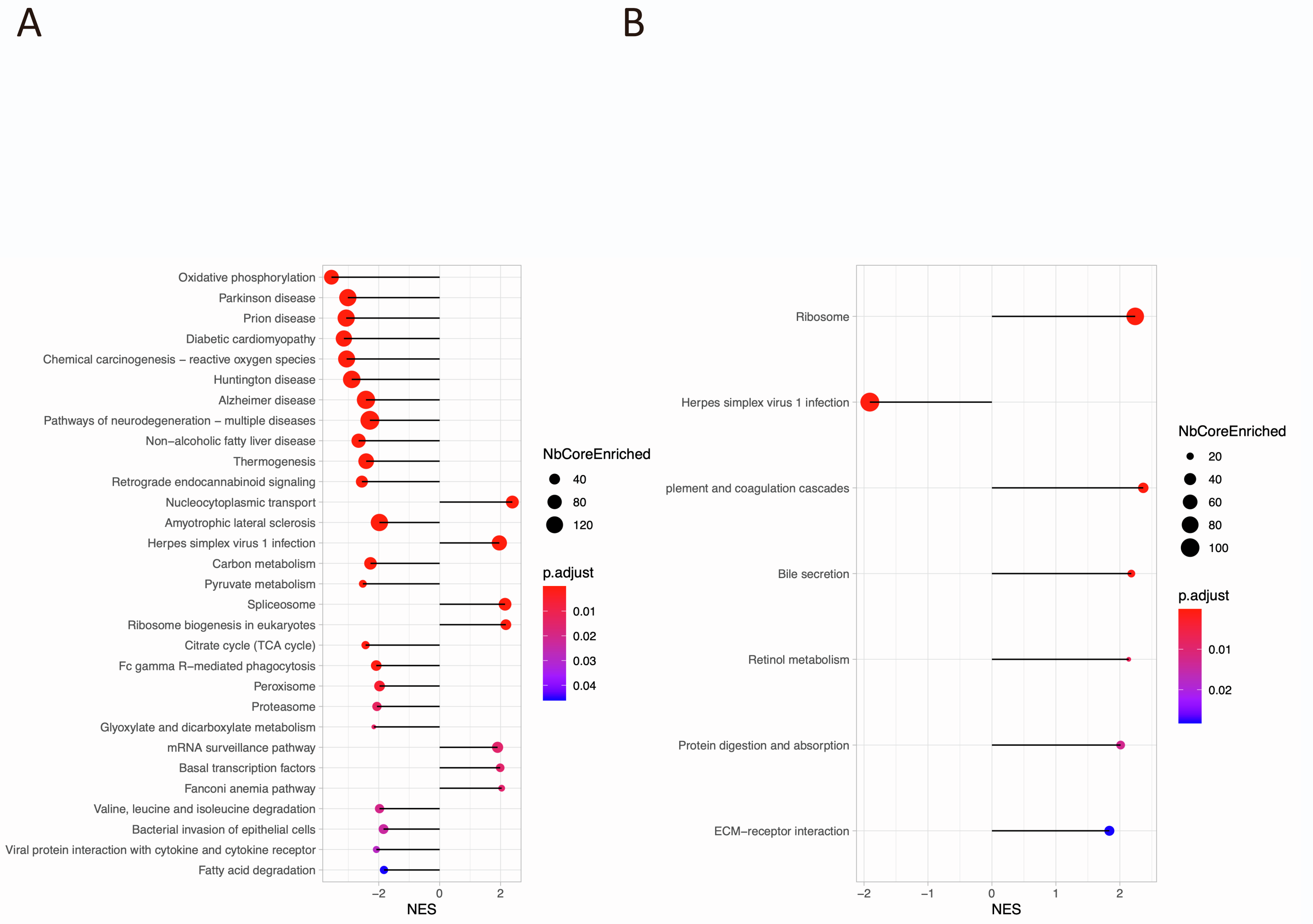

Figure S4: Muscle KEGG pathways regulated with glycemia in RC or HFD fed mice, related to Figure 4. A.) Plot showing the major KEGG pathways regulated with glycemia (adjusted p ≤ 0.05) in muscle from RC fed mice. B.) Plot showing the major KEGG pathways regulated with glycemia (adjusted p ≤ 0.05) in muscle from HFD fed mice

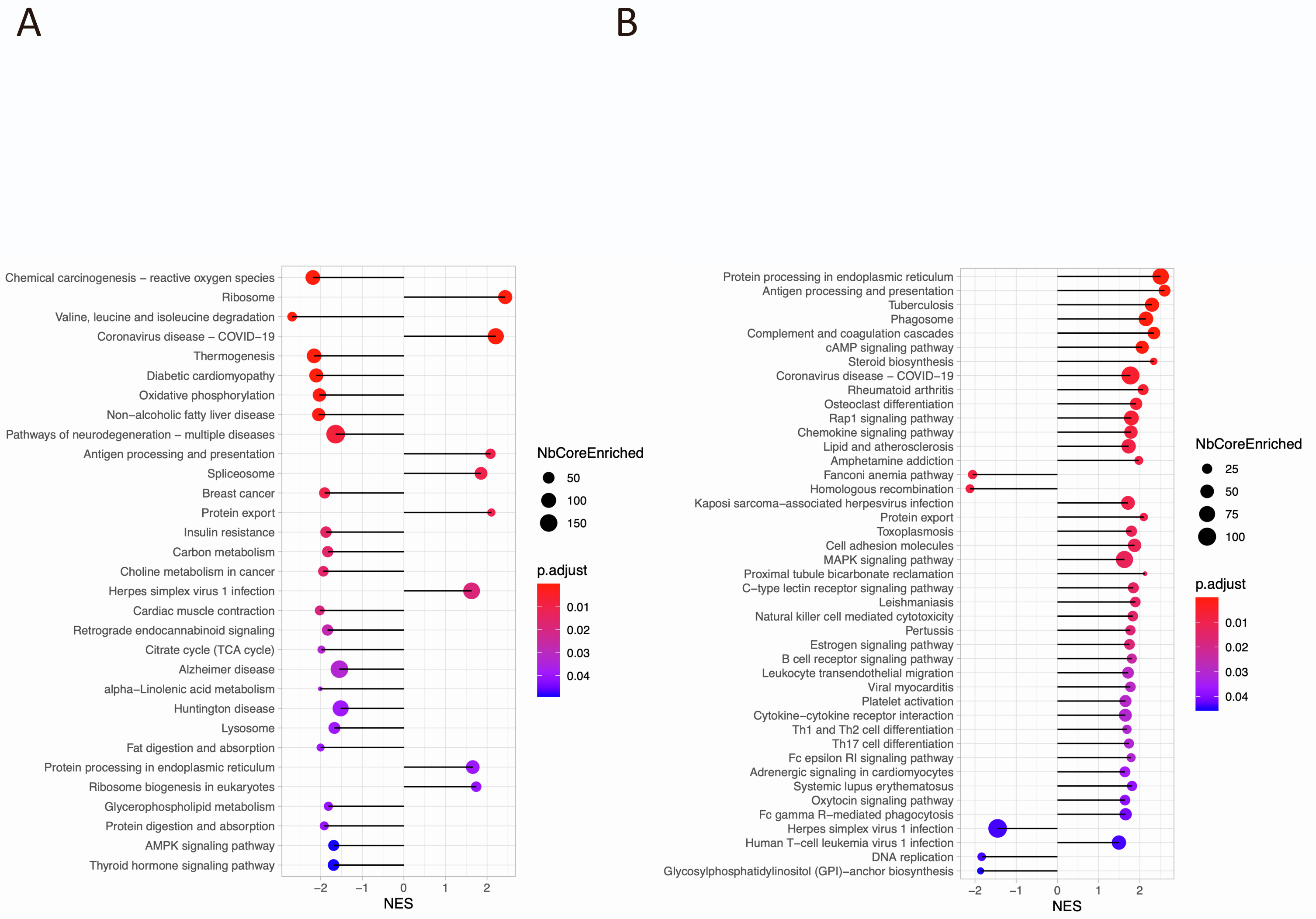

Figure S5: Liver KEGG pathways regulated with glycemia in RC or HFD fed mice, related to Figure 5. A.) Plot showing the major KEGG pathways regulated with glycemia (adjusted p ≤ 0.05) in liver from RC fed mice. B.) Plot showing the major KEGG pathways regulated with glycemia (adjusted p ≤ 0.05) in liver from HFD fed mice

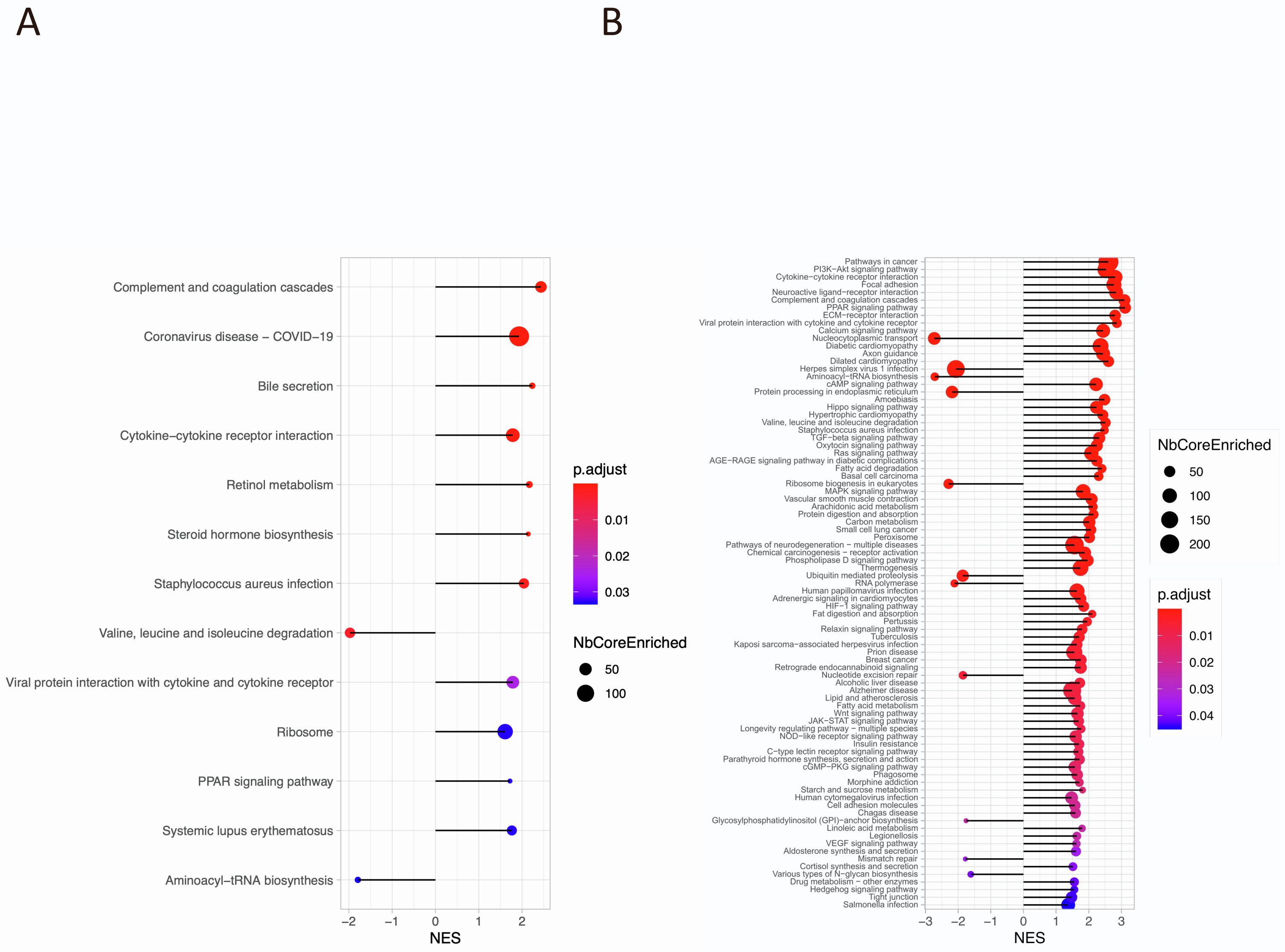

Figure S6: Visceral adipose KEGG pathways regulated with glycemia in RC or HFD fed mice, related to Figure 6. A.) Plot showing the major KEGG pathways regulated with glycemia (adjusted p ≤ 0.05) in adipose from RC fed mice. B.) Plot showing the major KEGG pathways regulated with glycemia (adjusted p ≤ 0.05) in adipose from HFD fed mice
